# Did smoking behavior change in adolescents and young adults with and without diabetes during the COVID-19 pandemic? A cohort study from the DPV registry

**DOI:** 10.1186/s12887-025-05434-w

**Published:** 2025-03-27

**Authors:** Katharina Warncke, Sabine E. Hofer, Simone von Sengbusch, Uwe Ermer, Mareike Niemeyer, Andreas Lemmer, Dörte Hilgard, Alena Welters, Reinhard W. Holl, Alexander J. Eckert

**Affiliations:** 1 Department of Pediatrics, Kinderklinik München Schwabing, Technical University of Munich School of Medicine, Kölner Platz 1, 80804 Munich, Germany; 2 Institute of Diabetes Research, Helmholtz Munich, German Center for Environmental Health, Munich, Germany; 3 Department of Pediatrics 1, Medical University of Innsbruck, Innsbruck, Austria; 4 Department of Paediatrics and Adolescent Medicine, University Hospital Schleswig-Holstein, Campus Luebeck, Luebeck, Germany; 5 St Elisabeth Klinik, Neuburg an der Donau, Germany; 6 Diabetes Centre for Children and Adolescents, Kinder- und Jugendkrankenhaus Auf der Bult, Hannover, Germany; 7 Department of Pediatrics and Adolescent Medicine, Helios Clinical Center, Erfurt, Germany; 8 Pediatric Endocrinology and Diabetology, Primary Psychosomatic Care, Witten, Germany; 9 Department of General Pediatrics, Neonatology and Pediatric Cardiology, Medical Faculty and University Hospital Düsseldorf, Heinrich Heine University Düsseldorf, Düsseldorf, Germany; 10 Institute of Epidemiology and Medical Biometry, University of Ulm, CAQM, Ulm, Germany; 11 German Center for Diabetes Research (DZD), Munich-Neuherberg, Germany

**Keywords:** Diabetes mellitus, Pandemic, Smoking, COVID-19

## Abstract

**Background:**

Smoking is a risk factor for cardiovascular complications and can promote a severe course of COVID-19 infection. The aim of this study was to compare smoking habits of young people with diabetes with the general population.

**Methods:**

We analyzed smoking behavior in the Diabetes Prospective Follow-up Registry (DPV) cohort (type 1 (T1D) and type 2 diabetes (T2D) from Germany and T1D from Austria aged 14–24 years) and compared it to data from the German survey on smoking behavior (DEBRA study) of the general population. Data were aggregated per year and patient for 2016–2023. Logistic regression models adjusted for gender and migration background were calculated stratified by age groups (14–17; 18–24 years), taking repeated measurements into account. Smoking behavior between T1D and T2D or between Germany and Austria was compared with similar regression models.

**Results:**

Thirty-four thousand two hundred seventy-five patients from the DPV cohort were included in data analysis. The overall proportion of people who smoked was lower in DPV than in the general population (13.4% vs. 24.0%), with the exception of young adults with T2D at the beginning of the pandemic (36.7% vs. 33.4%). For T1D, there was a significant upward trend in the number of patients who smoked in the group of 14–17 years (2.86%, CI 1.21–4.55 per year, *p* < 0.001) and also in the group of 18–24 years (4.94 per year, CI 1.37–8.63; *p* < 0.01) between 2016 and 2023. The proportion of smokers and the number of smoked cigarettes was higher in Austria than in Germany (10.7% vs. 8.0%; OR with 95%-CI 1.38 [1.22–1.56], *p* < 0.001; and 7.5 [6.8–8.1] vs. 5.9 [5.7–6.0] cigarettes/day, *p* < 0.001) and in T2D than T1D (11.0% vs. 7.9%; OR 1.44 [1.23–1.68], *p* < 0.001 and 8.0 [7.2–8.8] vs. 5.9 [5.7–6.1] cigarettes/day, *p* < 0.001).

**Conclusion:**

The reported proportion of smokers among young people with diabetes was lower than in the general population. Only young adults with T2D temporarily smoked more than the general population at the beginning of the pandemic. This could be explained by stress, but also by a changed daily structure during the lockdown.

**Supplementary Information:**

The online version contains supplementary material available at 10.1186/s12887-025-05434-w.

## Background

Smoking is a relevant risk factor for the development of cardiovascular complications in individuals with and without diabetes. Smoking is also associated with severe disease progression in COVID-19 infection [1]. A lower vaccination efficacy and higher infection rate among smokers are being discussed [2, 3]. In recent years, the COVID-19 pandemic and other factors like wars, global instability and climate thread and the resulting social consequences have increased the psychological burden particularly in the younger population. The pandemic has also been associated with changes affecting the cardiovascular risk profile of individuals with diabetes, such as a reduction in organized physical activity [4]. The “Deutsche Befragung zum Rauchverhalten” (DEBRA; German survey on smoking behavior) [5] collects data on smoking behavior in the general population and reported a percentage increase in smokers in the whole population in recent years. In addition to smoking, alcohol consumption has also been shown to have undergone changes during the pandemic, with an increase in consumption being particularly prevalent among vulnerable groups, such as those with depression and those with poor dietary quality [6, 7]. Due to general restrictions during the pandemic, such as lockdown, closure of playgrounds, schools and kindergardens, physical activity decreased and sedentary time increased predominantly among the younger population [8]. An increase in blood pressure and atherogenic lipid levels coinciding with a reduction in physical activity among young people with type 1 diabetes (T1D) during the COVID-19 pandemic was already shown in our Diabetes Prospective Follow-up Registry (Diabetes Patienten Verlaufsdokumentation, DPV) cohort [4]. In the current analysis, we aim to compare smoking behavior in the general population using data from the DEBRA study with that of young people with diabetes using data from the DPV registry and to analyze differences between countries (Germany and Austria), sex and diabetes type.

## Research design and methods

### Study design and participants

This observational study is based on data from the DPV registry. In the DPV registry, pseudonymous standardized, prospective data from routine diabetes care are transmitted by diabetes centers from Germany, Austria, Switzerland, and Luxembourg to Ulm University for central validation and benchmarking analysis twice a year. For optimal data validity, inconsistent data are reported back to participating centers, corrected if necessary, and re-entered into the database as previously described [9]. Analysis of anonymized data within the DPV initiative was approved by the Ethics Committee of the Medical Faculty of the University of Ulm, Germany, and the institutional review boards at the participating centers.

For this analysis, the data set of March 2024 was used, including 346 centers from Germany and Austria which contributed data to this analysis. Due to the low number of cases, patients from Switzerland and Luxembourg were not included in this analysis.

**Fig. 1:**
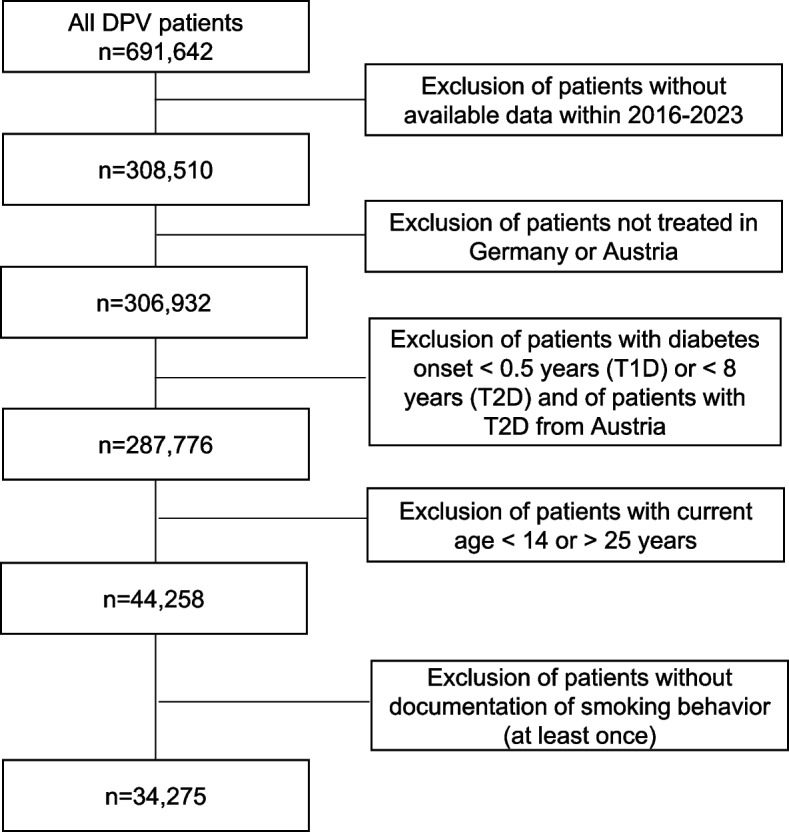
Inclusion and exclusion criteria. DPV: Diabetes Prospective Follow-up Registry (Diabetes Patienten Verlaufsdokumentation). T1D: type 1 diabetes; T2D: type 2 diabetes

We evaluated patients with documented data within the time period from 2016 to 2023. From Germany patients with T1D and T2D were included, from Austria only patients with T1D were analyzed. The capture rate of adolescent T2D patients in Austria within DPV is too low, to include these data in the analysis. Patients with a clinical diagnosis of T1D were only included if they were ≥ 6 months of age at diabetes onset and patients with T2D were only included if they were ≥ 8 years at diagnosis as a diagnosis of T2D is very unlikely in children under the age of 8 years. After exclusion of patients without available data within 2016–2023, patients with diabetes onset < 6 months of age (T1D) or < 8 years of age (T2D), with other diabetes types than T1D/T2D and/or from other countries than Germany/Austria, 44,258 patients aged 14–24 years remained for data analysis. Of these, we only included patients with documented data on smoking behavior at least once, resulting in 34,275 patients from the DPV registry (Fig. 1).

Smoking frequency and proportion of people who smoked were compared between patients with T1D and T2D from the DPV registry and adolescents and young adults from the DEBRA study as a representative cohort for the general German population. The DEBRA study has been collecting data on smoking behavior among people > 14 years of age since 2016 based on computer-assisted, face-to-face household interviews. From 2016 to now, > 100,000 people were surveyed [5, 10, 11]. Furthermore, we examined the number of cigarettes smoked per year, diabetes type, country, age group and depending on whether migration background was reported as well as differences in demographic, clinical, laboratory and other characteristics between people with diabetes who smoked and people who did not smoke within the DPV registry.

### Data

Smoking was defined as cigarette smoking of at least one cigarette per day. E-cigarettes, other smokable substances or oral nicotine products like snus were not included. Hemoglobin A1c (A1c) was used as an indicator of glycemic control. Levels were mathematically standardized to the Diabetes Control and Complications Trial (DCCT) reference range of 4.05–6.05% with the MOM (multiple of mean) transformation to correct for different laboratory methods used by study centers [12]. Anthropometric measurements were performed in the local centres according to inhouse protocols and analyzed using contemporary German reference data for height and weight (Arbeitsgemeinschaft Adipositas im Kindes- und Jugendalter, AGA) [13]. These reference data were used for patients from Germany and Austria. The BMI (body mass index: weight in kilograms/(height in meters)^2^), is an accepted measure of overweight and obesity in children, adolescents and young adults. BMI-SDS (standard deviation score) values were generated using the LMS method [13–15]. Migration background was defined as the patient or one of his/her parents born outside of Germany/Austria. The percentage of patients with insulin therapy, use of a continuous glucose monitoring system (CGMS), sensor augmented pump therapy and a hybrid closed loop system was described in relation to the respective cohort, calculated individually for smokers and non-smokers and compared statistically between the two groups.

### Statistical analysis

Data were analyzed using SAS 9.4 (TS1M7, SAS Institute, Cary, NC). If data for a specific variable were not available in individual cases, the case was not considered for the analysis of that variable, except for migration background where missing data were considered as no migration background. Descriptive analyses with median and interquartile ranges (IQR) for continuous variables and numbers (proportions) for binary variables are presented for age, current anthropometric characteristics and A1C in the most recent treatment year of each patient.

Data on the proportion of smokers in the general population were taken from the DEBRA website [11] as aggregated data per year and age group (14–17 and 18–24 years). Therefore, no confidence intervals (CI) could be presented for DEBRA data.

The DPV data on smoking habits were aggregated per year and patient for 2016–2023. Logistic regression models for the proportion of smokers and linear regression models for the number of cigarettes/day among smokers were calculated and stratified for age groups (14–17 and 18–24 years) and adjusted for gender and migration background, taking repeated measures into account. Similar models were implemented to calculate the proportion of smokers or the number of cigarettes/day over the years 2016–2023 between the countries (only for T1D), diabetes types (only Germany), sex and migration background (both stratified by country and diabetes type).

## Results

Thirty-four thousand two hundred seventy-five patients from the DPV registry were included in the analysis (Fig. 1). Descriptive clinical and laboratory data of the cohort, divided into T1D (Germany/Austria) and T2D (Germany only) and referring to the most recent year of treatment, are summarized in Table 1. An overview regarding the differences (unadjusted comparison) between patients who smoked and those who did not smoke is displayed in Table 2. Modern therapies such as insulin pumps, CGMS, sensor augmented pump therapy and hybrid closed loop systems were used more frequently by non-smoking patients than by smoking patients. This was significant in the group of patients with T1D from Germany (Table 2, unadjusted comparison).

**Table 1:** Clinical and laboratory data in patients with T1D (Germany and Austria) and T2D (Germany); the numbers refer to the most recent year of treatment

	**n**	**Patients with T1D (Germany)**	**n**	**Patients with T1D (Austria)**	**n**	**Patients with T2D (Germany)**
Total number of patients		30,998		2,025		1,252
Current age (yrs)	30,998	17.5 (16.2–18.5)	2,025	17.8 (16.8–19.0)	1,252	17.4 (16.0–19.6)
Diabetes duration (yrs)	30,998	7.3 (3.9–11.2)	2,025	8.3 (4.7–12.1)	1,252	1.4 (0.2–3.1)
Sex (% male)	30,998	54.5	2,025	56.1	1,252	43.2
Migration background (((%)	30,998	22.7	2,025	28.7	1,252	33.0
Current BMI SDS^a^	27,940	0.6 (−0.1–1.3)	1,787	0.6 (−0.1–1.3)	1,108	2.6 (2.1–3.1)
Current A1c (%)	29,787	7.8 (7.0–8.8)	1,962	7.7 (6.9–8.7)	971	6.5 (5.6–8.1)
Current A1c (mmol/mol)	29,787	61.2 (52.6–73.1)	1,962	60.4 (52.2–72.0)	971	47.2 (37.9–64.8)
% of smokers	30,998	13.1	2,025	15.9	1,252	16.9
% of patients physically activity (≥ once/week)	24,322	64.6	1,525	74.1	879	49.7
% with elevated blood pressure	30,544	10.7	1,797	12.1	1,220	27.8
% with dyslipidemia	23,116	28.4	1,574	19.2	859	63.7
% with insulin therapy	30,998	100	2,025	100	1,252	38.0
% with insulin pump therapy	30,998	49.4	2,025	55.1	1,252	1.2
% with CGMS	30,998	73.3	2,025	77.0	1,252	22.0
% with sensor-augmented pump therapy	30,998	39.6	2,025	44.0	1,252	0.6
% with hybrid closed loop system	30,998	15.2	2,025	15.4	1,252	0.4

Values are expressed as medians with interquartile ranges [25th–75th percentile] or as percentage of cases. A1c, hemoglobin A1c. CGMS, continuous glucose monitoring system

^a^SDS calculated using national reference data (AGA) [13]

**Table 2:** Comparison between smokers and non-smokers by type of diabetes and country of origin (unadjusted data); the numbers refer to the most recent year of treatment

	**Patients with T1D (Germany)**			**Patients with T1D (Austria)**			**Patients with T2D (Germany)**		
	**non-smoking**	**smoking**	***p***	**non-smoking**	**smoking**	***p***	**non-smoking**	**smoking**	***p***
**Total n**	26,947	4,051		1,704	321		1,040	212	
**Age (yrs)**	17.4 (16.0–18.3) [26,947]	17.9 (17.3–20.1) [4,051]	< 0.00001	17.8 (16.5–19.0) [1,704]	18.1 (17.6–19.4) [321]	< 0.00001	17.2 (15.8–18.8) [1,040]	18.3 (17.2–22.6) [212]	< 0.00001
**Diab. duration (yrs)**	7.2 (3.8–11.1) [26,947]	8.1 (4.5–12.0) [4,051]	< 0.00001	8.3 (4.6–12.2) [1,704]	8.4 (5.1–12.0) [321]	n.s	1.3 (0.3–3.1) [1,040]	1.7 (0.2–3.2) [212]	n.s
**Sex (% male)**	53.7 [26,947]	59.9 [4,051]	< 0.00001	55.6 [1,704]	58.9 [321]	n.s	43.6 [1,040]	41.5 [212]	n.s
**Migration background**	23.2 [26,947]	19.4 [4,051]	< 0.00001	29.1 [1,704]	26.8 [321]	n.s	35.1 [1,040]	22.6 [212]	< 0.01
**BMI-SDS**^**a**^	0.6 (−0.1–1.3) [24,676]	0.6 (−0.2–1.3) [3,264]	< 0.01	0.6 (−0.1–1.3) [1,505]	0.6 (−0.1–1.4) [282]	n.s	2.6 (2.1–3.1) [934]	2.6 (2.0–3.1) [174]	n.s
**Current A1c (%)**	7.6 (6.9–8.6) [25,897]	8.8 (7.7–10.3) [3,890]	< 0.00001	7.6 (6.9–8.5) [1,648]	8.7 (7.5–10.0) [314]	< 0.00001	6.4 (5.6–7.8) [813]	7.1 (5.9–9.5) [158]	< 0.0001
**A1c (mmol/mol)**	59.9 (51.8–70.5) [25,897]	73.1 (60.1–89.0) [3,890]		59.3 (51.3–69.2) [1,648]	71.0 (58.2–85.3) [314]		46.0 (37.2–61.7) [813]	54.2 (40.9–80.6) [158]	
**% of patients with physical activity**^**b**^	66.1 [21,947]	54.4 [3,049]	< 0.00001	77.7 [1,271]	55.9 [254]	< 0.00001	51.2 [746]	41.4 [133]	n.s
**% with elevated blood pressure**	10.6 [26,559]	11.4 [3,985]	n.s	12.2 [1,502]	11.5 [295]	n.s	27.1 [1,014]	31.1 [206]	n.s
**% with dyslipidemia**	27.3 [20,116]	35.7 [3,000]	< 0.00001	18.0 [1,325]	25.3 [249]	n.s	61.5 [699]	73.1 [160]	n.s
**% of patients with insulin therapy**^**c**^	n.a	n.a	n.a	n.a	n.a	n.a	37.5 [1,040]	40.6 [212]	n.s
**% with insulin pump therapy**	50.4 [26,947]	42.6 [4,051]	< 0.00001	55.9	51.1	n.s	n.a	n.a	n.a
**% with CGMS**	74.8 [26,947]	63.7 [4,051]	< 0.00001	77.2	76.0	n.s	n.a	n.a	n.a
**% with sensor-augmented pump therapy**	40.8 [26,947]	31.9 [4,051]	< 0.00001	45.2	37.7	n.s	n.a	n.a	n.a
**% with hybrid closed loop system**	15.9 [26,947]	10.1 [4,051]	< 0.00001	16.4	9.7	< 0.05	n.a	n.a	n.a

Values are expressed as medians with interquartile ranges [25th–75th percentile] or as percentage of cases. A1c, hemoglobin A1c. CGMS, continuous glucose monitoring system

^a^SDS calculated using national reference data (AGA) [13]

^b^means physical activity ≥ once a week: the number of patients available for each variable is shown in square brackets in each cell

^c^The proportion of patients on insulin therapy was only compared between smokers and non-smokers in patients with T2D, as all patients with T1D were treated with insulin, regardless of whether they smoked or not

### Development of smoking behavior before, during and after the COVID-19 pandemic

**Fig. 2:**
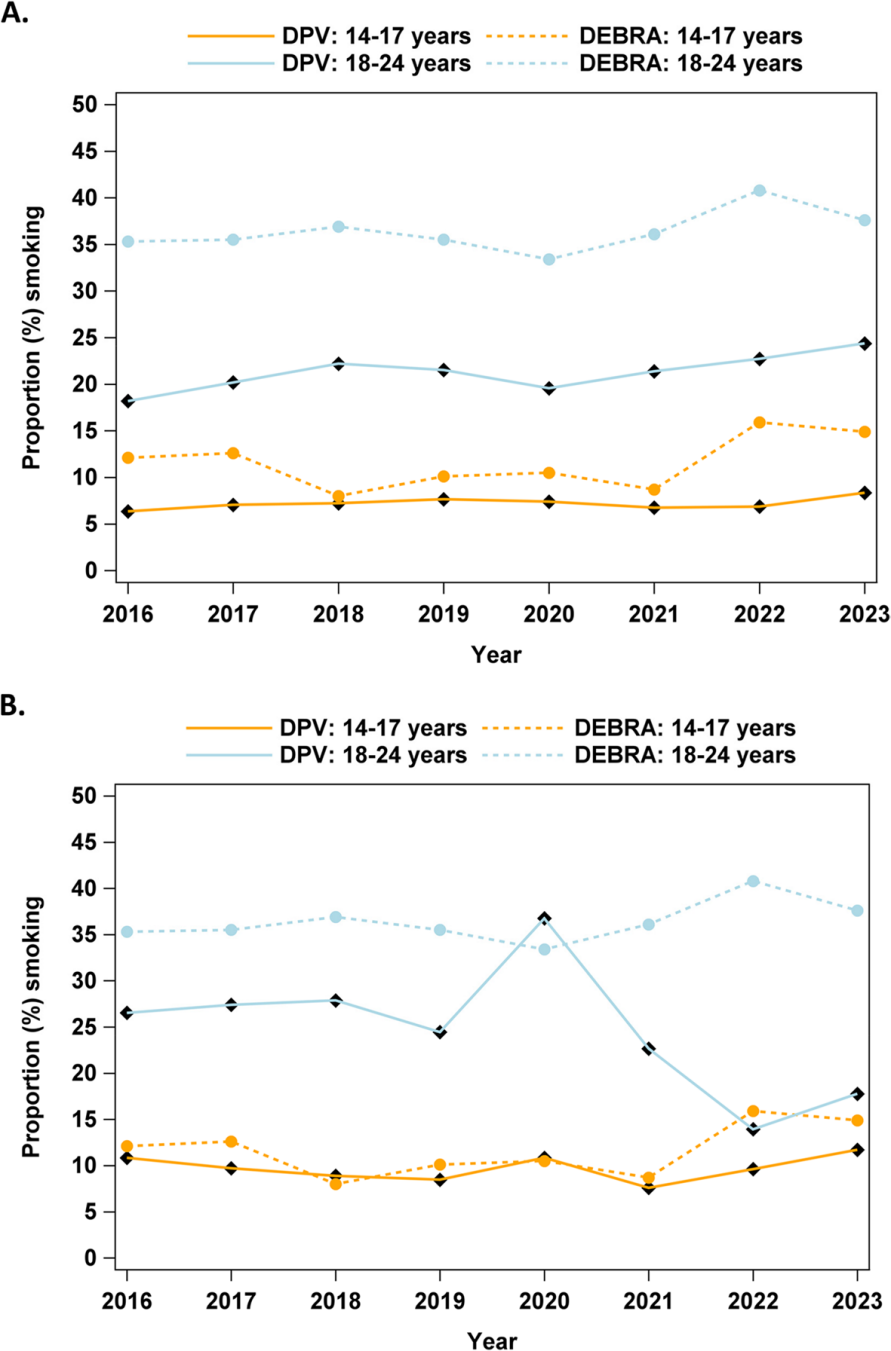
**a** Proportion of smokers among individuals with type 1 diabetes (T1D) in the DPV registry vs. results from the DEBRA study (general population) from 2016 to 2023. DPV: Diabetes Prospective Follow-up Registry (Diabetes Patienten Verlaufsdokumentation). **b** Proportion of smokers among individuals with type 2 diabetes (T2D) in the DPV registry vs. results from the DEBRA study (general population) from 2016 to 2023. DPV: Diabetes Prospective Follow-up Registry (Diabetes Patienten Verlaufsdokumentation)

The proportion of smokers over the whole period from 2016–2023 was lower in the DPV cohort than in the general population averaged over all survey periods of the DEBRA study. In particular, smoking was reported in 13.4% of the whole analysed DPV cohort, in 13.1% of T1D, 16.9% with T2D (Germany) and 15.9% with T1D (Austria) vs. 24.0% in the general population (DEBRA); Fig. 2. Solely, at the outset of the pandemic in 2020, a higher proportion of young adults with T2D reported smoking than was the case for the general German population. Indeed, the proportion of smokers among this cohort was 36.7% compared to 33.4% in the DEBRA study (Table 3 and Fig. 2b). In the younger group with T1D aged 14–17 years, the proportion of smokers was 6.4% in 2016 and 8.3% in 2023 after adjusting for sex, migration background, diabetes duration and taking repeated measurements into account, with a slightly increasing trend (relative increase: 2.86%, CI 1.21–4.55 per year, *p* < 0.001). In the older age group (18–24 years), the adjusted percentage of patients who reported smoking increased from 18.2% in 2016 to 22.2% in 2018, dropped to 19.6% in 2020 and increased again to the highest value of 24.4% in 2023, with an overall relative increase between 2016 and 2023 of 4.94%, CI 1.37–8.63, *p* < 0.01 (Fig. 2a). Among younger patients with T2D, the percentage of smokers ranged between 7.6 and 11.7% (Table 3), while among young adults with T2D, the percentage of smokers was very high at 36.7% in 2020 (Table 3 and Fig. 2b). However, for T2D patients, no significant trends for smoking behavior could be observed from 2016 to 2023.

**Table 3:** Raw and adjusted percentages (adjusted for sex, migration background, diabetes duration; taking repeated measurements into account) of smokers among the DPV cohort by age, T1D, T2D in the different years from 2016–2023 and percentages of smokers from the general population (DEBRA study)

	**Patients aged 14–17 years**					**Patients aged 18–24 years**				
	**T1D raw**	**T1D adjusted**	**T2D raw**	**T2D adjusted**	**DEBRA-Study**	**T1D raw**	**T1D adjusted**	**T2D raw**	**T2D adjusted**	**DEBRA-Study**
**2016**	7.9	6.4	11.8	10.8	12.1	17.4	18.2	28.6	26.5	35.3
**2017**	8.0	7.1	10.5	9.7	12.6	19.5	20.2	30.1	27.4	35.5
**2018**	7.7	7.2	9.3	8.9	8.0	22.6	22.2	28.2	27.9	36.9
**2019**	7.9	7.7	8.2	8.5	10.1	21.2	21.5	30.5	24.4	35.5
**2020**	7.4	7.4	10.1	10.8	10.5	19.7	19.6	42.9	36.7	33.4
**2021**	6.4	6.7	7.5	7.6	8.7	20.8	21.4	21.7	22.7	36.1
**2022**	6.2	6.9	8.3	9.6	15.9	22.0	22.7	12.8	13.9	40.8
**2023**	7.1	8.3	10.2	11.7	14.9	23.8	24.4	18.9	17.8	37.6

**Fig. 3:**
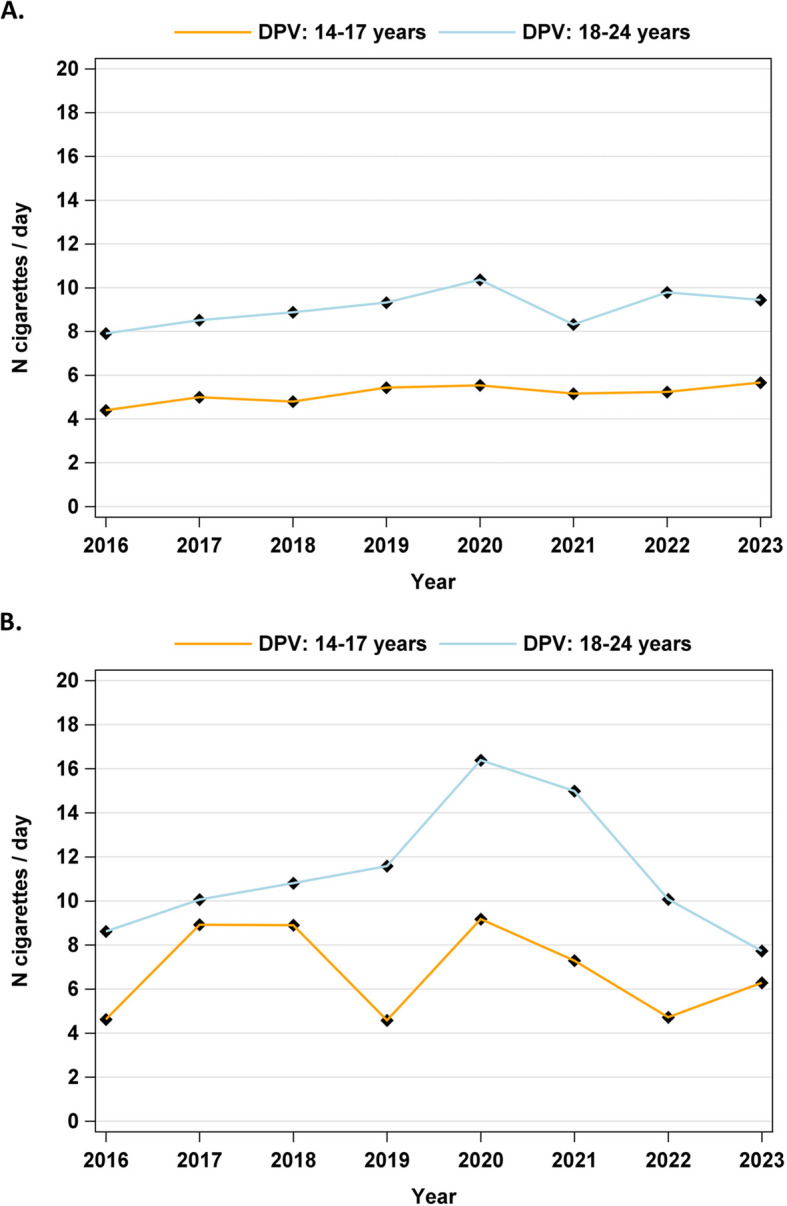
**a** Number of cigarettes/day among smokers with type 1 diabetes (T1D) from 2016 to 2023. DPV: Diabetes Prospective Follow-up Registry (Diabetes Patienten Verlaufsdokumentation).** b** Number of cigarettes/day among smokers with type 2 diabetes (T2D) from 2016 to 2023. DPV: Diabetes Prospective Follow-up Registry (Diabetes Patienten Verlaufsdokumentation)

Both the younger (14–17 years; 0.14 cigarettes per day and year, CI 0.05–0.23, *p* < 0.001) and the older (18–24 years; 0.27 cigarettes per day and year, CI 0.08–0.46, *p* < 0.01) age groups with T1D showed a significant increasing trend for smoked cigarettes per day between 2016 and 2023, which was more pronounced in the older age group (Table 4 and Fig. 3a). For patients with T2D, there were fluctuations with a higher number of cigarettes smoked per day at the beginning of the pandemic in 2020, especially in the older age group, but there were no significant trends, (Table 4 and Fig. 3b).

**Table 4:** Estimated numbers of smoked cigarettes/day among smokers from the DPV cohort (Germany) in the different years from 2016–2023 adjusted for sex and migration background

	**Patients aged 14–17 years**		**Patients aged 18–24 years**	
	**T1D**	**T2D**	**T1D**	**T2D**
**2016**	4.4 (4.0–4.8)	4.6 (1.3–8.0)	7.9 (7.3–8.5)	8.6 (5.6–11.6)
**2017**	5.0 (4.6–5.4)	8.9 (5.8–12.1)	8.5 (7.9–9.1)	10.1 (7.1–13.0)
**2018**	4.8 (4.4–5.2)	8.9 (5.7–12.1)	8.9 (8.2–9.6)	10.8 (8.0–13.6)
**2019**	5.4 (5.0–5.8)	4.6 (1.3–7.9)	9.3 (8.5–10.2)	11.6 (8.4–14.8)
**2020**	5.5 (5.1–5.9)	9.2 (6.2–12.1)	10.4 (9.2–11.5)	16.4 (13.1–19.7)
**2021**	5.2 (4.7–5.6)	7.3 (4.3–10.3)	8.3 (7.1–9.5)	15.0 (10.7–19.2)
**2022**	5.2 (4.8–5.7)	4.7 (1.9–7.6)	9.8 (8.4–11.2)	10.1 (4.2–15.9)
**2023**	5.7 (5.2–6.1)	6.3 (3.6–9.0)	9.4 (8.0–10.9)	7.7 (2.3–13.2)

Data are presented as mean values with lower and upper confidence intervals

### Adjusted differences of smoking by country, sex and diabetes type

The percentage of patients with T1D who smoked was higher in Austria than in Germany (10.7% vs. 8.0%; OR with 95%-CI 1.38 [1.22–1.56], *p* < 0.001) and the number of smoked cigarettes/day with 95%-CI among smokers was also higher in Austria (7.5 [6.8–8.1] vs. 5.9 [5.7–6.0] cigarettes/day, *p* < 0.001)). Comparing patients with T1D and T2D from Germany, smoking was more frequent in patients with T2D than in those with T1D (11.0% vs. 7.9%; OR 1.44 [1.23–1.68], *p* < 0.001), and also the number of cigarettes/day was higher in T2D than in T1D patients (8.0 [7.2–8.8] vs. 5.9 [5.7–6.1] cigarettes/day, *p* < 0.001). Among T1D patients from Germany, the frequency of smoking was higher in males than in females (8.7% vs. 7.0%; OR 1.27 [1.19–1.36], *p* < 0.001), and also the number of smoked cigarettes was higher in males than in females (6.2 [6.0–6.5] vs. 5.4 [5.1–5.6], *p* < 0.001). Among patients with T1D from Austria, the male predominance for smoking (11.2% vs. 11.0%; OR 1.03 [0.80–1.31], *p* = 0.841) and for the number of cigarettes smoked per day (7.8 [6.8–8.8] vs. 6.7 [5.5–7.8], *p* = 0.143) was not significant. No gender difference at all was observed for the proportion who smoked among T2D patients (males and females 12.0%; OR 1.01 [0.74–1.37], *p* = 0.974); moreover, the number of cigarettes smoked among smokers was not significantly higher among men than women (9.3 [8.0–10.8] vs. 8.4 [7.2–9.7], *p* = 0.386). Among T1D patients from Germany, the frequency of smoking was slightly higher in patients without migration background than in patients who reported migration background (8.1% vs. 7.2%; OR 1.15 [1.06–1.25], *p* < 0.01), while there was no significant difference regarding the number of cigarettes smoked (5.8 [5.6–6.0] vs. 6.0 [5.6–6.4], *p* = 0.370). Among patients with T2D, there was no significant difference between patients with and without migration background, neither for the proportion of smokers (13.2% for patients without migration background vs. 10.2% for patients with migration background; OR 1.34 [0.92–1.96], *p* = 0.126) nor for the number of smoked cigarettes among smokers (8.7 [7.6–9.9] vs. 9.0 [6.8–11.1], *p* = 0.850).

## Discussion

We showed that young individuals with diabetes report smoking less frequently compared to the general German population, with the exception of young adults with T2D at the beginning of the COVID-19 pandemic. The reasons for this observation may be manifold. Children with a chronic disease grow up with their parents controlling their health behavior longer and more intensively. They regularly attend a special consultation where risky behavior, including smoking, is discussed from puberty onwards. Presumably this increases awareness of the negative effects of smoking, especially in combination with diabetes.

The difference in the proportions of smokers between persons with diabetes and from the general population was more pronounced in the older age group (18–24 years) than in the younger age group (14–17 years), which is presumably due to the fact that the younger age group smokes significantly less overall. Possible reasons for this are that, according to the law, the younger age group is not yet allowed to buy cigarettes (permitted from the age of 18), has less money available for consumption as schoolchildren and is controlled even more closely by their parents. However, there are also factors that could have contributed to falsely low rates: Children and adolescents are often accompanied by a parent at medical appointments, which could lead to false information about smoking behavior. However, a trusting relationship with the diabetologist helps a lot to ensure that questions about smoking behavior are answered honestly, even in the presence of the parents. The self-reports of children and adolescents documented in the DPV register could have been influenced by a lack of questioning on the part of the doctor and a deliberately desired (negative) response from the adolescents.

The proportion of smokers among the younger age group with T1D is similar to that in Italy with a proportion of 10% among T1D patients < 20 years [16], but for example higher than the reported proportion of a study from Turkey (1%), [17]. In the USA, a percentage of 20–25% smokers was reported among adolescents and young adults with T1D and T2D, which was higher than in the general population [18–20]. This is discordant with our findings of a lower percentage of smokers among people with T1D and T2D.

The observation of a high proportion of smokers among patients with T2D in 2020 followed by decreasing proportion in the years 2021 and 2022 could be explained by the fact that more people initially smoked as a result of the psychological burden caused by the pandemic, but also due to more opportunities to smoke, such as working from home or online-schooling. After it became known that smoking may promote a severe course of COVID-19 [21], many young people may probably have stopped smoking again. An increase in health awareness combined with broadly spread information about unfavourable effect of smoking in COVID-19 infection might have led to smoking cessation. Furthermore, it is possible that less participation in social events with smoking participants took place. Another explanation would be that at the beginning of the pandemic, there were significantly fewer outpatient appointments and then patients with many problems, including smoking, were more likely to be seen in diabetes consultations than other patients. In principle, it is conceivable that there could be a selection bias, as it is possible that more severely affected patients with T2D were more likely to consult a doctor during the pandemic. These patients may in turn have been more likely to smoke. However, it is more plausible that the pandemic has caused a temporary change in social behavior and lifestyle particularly in the group of subjects with T2D. The association of smoking with T2D is well studied and may be related to changes in health awareness and lifestyle factors, and T2D could be associated with an unfavourable lifestyle in general [22]. Consistent with this, we identified a higher proportion of individuals with cardiovascular risk factors in T2D patients than in T1D patients and among T2D patients, cardiovascular risk factors were more frequently seen in patients who smoked. During the pandemic, an increase in cardiovascular risk factors such as lack of physical activity or hypertension was also described in patients with T1D in the DPV cohort [4]. However, as our data show, these factors were even more pronounced in the cohort with T2D.

Another possible explanation for the higher percentage of smokers among young people with T2D compared to T1D could be that people with T2D might see smoking as an opportunity to lose weight, as smoking leads to an inhibition of appetite [23]. Both the percentage of smokers and the number of cigarettes smoked were slightly higher in Austria than in Germany. The higher percentage of smokers in Austria could be due to the fact that the sale of tobacco products to 16–18 year olds was still permitted until the beginning of 2019, and the age limit for sales was only raised to 18 years in 2019, while this age limit has been established in Germany since 2007. Among patients with T1D in Germany, the proportion of men among smokers was higher, consistent with previous data from the DPV registry and the US T1DX registry [18], while for young people with T2D in Germany and for individuals with T1D from Austria, no significant difference between men and women was reported. In the general population of both countries, smoking is more common among men then among women, but gender differences have decreased over time [24, 25].

In recent decades, the negative impact of smoking on health has been clearly recognized worldwide and anti-smoking programs and cessation programs have been established, leading to varying degrees of decline in the proportion of smokers in different countries [26]. In this paper, we were able to show that this downward trend was broken or even reversed by the COVID-19 pandemic, especially among young adults.

In the DPV cohort, smoking was associated with a higher overall cardiovascular risk, including a higher percentage of patients with dyslipidemia, higher A1c levels and a lower rate of physical activity. Although the relationship between smoking and an elevated risk of cardiovascular disease has been extensively described [27–29], there is a paucity of evidence regarding the correlation between smoking and poor metabolism [18, 30], and more extensive research on this topic is certainly needed. The more frequent use of modern therapeutic options such as insulin pumps by non-smoking patients with T1D that we observed could be related to a change in health awareness among non-smokers. This association has already been described in the literature [31]. Further efforts must be made to encourage children, adolescents and young adults with and without diabetes to refrain from smoking.

Corresponding prevention programs for schools have been tested and established in many countries [32]. Anti-smoking campaigns in early adolescence might be more effective than smoking cessation programs in the long run. In this concept, it is important to mention that prevention in clinics (especially for T1D) may be more effective than prevention outside of clinics (probably for T2D), as diabetes teams are aware of the negative impact of smoking in people with T1D. People with T1D are in a long term monitoring and control with building close relationships with their diabetes teams. This might show greater impact in individual counseling and smoking prevention than preventive measures only through brochures or the media.

### Strengths and limitations

A strength of the DPV registry cohort is its inclusion of around 80–90% of pediatric patients with diabetes in Germany and Austria [33]. This allowed us to apply the analysis of smoking behavior to a large number of patients with diabetes with a relatively long follow-up time. The main limitations of our study is the self reporting on smoking habits and the exclusion of patients who did not report smoking. It is unclear whether they were not asked, did not answer, or whether the information was not documented.

### Conclusions

The proportion of self-reported/documented smokers is remarkably lower among adolescents and young adults with diabetes compared to the general population, which could be an effect of medical education, frequent clinical visits and medical advice given in this patient group. Still, a tremendous effort is needed to reduce smoking in both, the general population and among young people with diabetes. Previous efforts to reduce smoking among the population have been at least temporarily and partially thwarted by the COVID-19 pandemic.

## Supplementary Information

Supplementary Material 1.

## Data Availability

AJE had full access to all the data in the study and takes responsibility for the integrity of the data and the accuracy of the data analysis. The aggregated datasets used and/or analyzed during the current study can be requested from PD Dr. Stefanie Lanzinger (University of Ulm, Institute of Epidemiology and Medical Biometry, Ulm, Germany; stefanie.lanzinger@uni-ulm.de). Because of patient protection and the agreements provided by the patient, data on the individual participant level cannot be shared with collaborators; however, remote data analysis is possible.

## Abbreviations

A1c: Hemoglobin A1c
AGA: Arbeitsgemeinschaft Adipositas im Kindes- und Jugendalter
BMI: Body Mass Index
CGMS: Continuous glucose monitoring system
CI: Confidence interval
DCCT: Diabetes Control and Complications Trial
DEBRA: Deutsche Befragung zum Rauchverhalten (German survey on smoking behavior)
DPV: Diabetes Patienten Verlaufsdokumentation (Diabetes Prospective Follow-up Registry)
IQR: Interquartile range
MOM: Multiple of mean
SDS: Standard deviation score
T1D: Type 1 diabetes
T2D: Type 2 diabetes
