# Supplementary material for: Did smoking behavior change in adolescents and young adults with and without diabetes during the COVID-19 pandemic? A cohort study from the DPV registry
Source: BMC Pediatr. 2025 Mar 27;25:236. doi: 10.1186/s12887-025-05434-w (PMC11948826; doi:10.1186/s12887-025-05434-w)
Supplement: Supplementary file 1 — Supplementary Material 1. [file 12887_2025_5434_MOESM1_ESM.docx]

**List of DPV centers contributing to this analysis**

Aachen - Innere RWTH, Aachen - Uni-Kinderklinik RWTH, Aalen Kinderklinik, Ahlen St. Franziskus Kinderklinik, Altötting Kinderklinik Zentrum Inn-Salzach, Amberg Kinderklinik St. Marien, Arnsberg-Hüsten Karolinenhosp. Kinderabteilung, Asbach Kamillus-Klinik Innere, Aue Helios Kinderklink, Augsburg IV. Med. Uni-Klinik, Augsburg Josefinum Kinderklinik, Augsburg Uni-Kinderklinik, Aurich Kinderklinik, Bad Aibling Internist. Praxis, Bad Driburg / Bad Hermannsborn Innere, Bad Hersfeld Innere, Bad Hersfeld Kinderklinik, Bad Kreuznach Diakonie Kikli, Bad Kreuznach-Viktoriastift, Bad Kösen Median Kinderklinik, Bad Mergentheim - Diabetesfachklinik, Bad Mergentheim - Kinderdiabetologische Praxis, Bad Oeynhausen Herz-und Diabeteszentrum NRW, Bad Orb Spessart Klinik, Bad Reichenhall Kreisklinik Innere Med., Bad Salzungen Kinderklinik, Bautzen Oberlausitz KK, Bayreuth Innere Medizin, Berchtesgaden CJD, Berchtesgaden CJD-Beruf.REHA, Bergen Gemeinschaftspraxis, Berlin DRK-Kliniken Mitte Innere, Berlin DRK-Kliniken Pädiatrie, Berlin Klinik St. Hedwig Innere, Berlin Lichtenberg - Kinderklinik, Berlin Oskar Zieten Krankenhaus Innere, Berlin Parkklinik Weissensee, Berlin Schlosspark-Klinik Innere, Berlin Virchow-Kinderklinik, Berlin Vivantes Hellersdorf Innere, Bielefeld Kinderklinik Gilead, Bielefeld Pädiatrisches Forum, Bocholt Kinderklinik, Bochum Universitätskinderklinik St. Josef, Bodnegg - MVZ Wollmarshöhe, Bonn Schwerpunktpraxis, Bonn Uni-Kinderklinik, Bottrop Knappschaftskrankenhaus Innere, Braunschweig Kinderarztpraxis, Bremen - Kinderklinik Nord, Bremen - Mitte Innere, Bremen Eltern-Kind-Zentrum, Klinikum Mitte, Bremerhaven Kinderklinik, Bruchweiler Edelsteinklinik Kinder-Reha, Böblingen Kinderklinik, Castrop-Rauxel Evangelisches Krankenhaus, Castrop-Rauxel Rochus-Hospital, Celle Kinderarztpraxis, Celle Klinik für Kinder- und Jugendmedizin, Chemnitz Kinderklinik, Chemnitz-Hartmannsdorf Innere Medizin - DIAKOMED-1, Coburg Innere Medizin, Coburg Kinderklinik, Coesfeld Kinderklinik, Coesfeld/Dülmen Innere Med., Darmstadt Innere Medizin, Darmstadt Kinderklinik Prinz. Margaret, Datteln Vestische Kinderklinik, Deggendorf Medizinische Klinik II, Deggendorf Pädiatrie-Praxis, Delmenhorst JHD Kinderklinik, Dessau Kinderklinik, Dessau amb. Kinderarztzentrum, Detmold Kinderklinik, Dinslaken Kinderklinik, Dornbirn Innere Medizin, Dornbirn Kinderklinik, Dortmund Johannes Hospital, Dortmund Kinderklinik, Dortmund Knappschaftskrankenhaus Innere, Dortmund Medizinische Kliniken Nord, Dortmund-St. Josefshospital Innere, Dortmund-West Innere, Dresden Neustadt Kinderklinik, Dresden Uni-Kinderklinik, Duisburg Homberg Helios Rhein-Ruhr Kliniken GmbH, Duisburg Sana Kinderklinik, Duisburg St. Anna Innere Helios Rhein-Ruhr Kliniken GmbH, Duisburg-St.Johannes Helios, Düren-Birkesdorf Kinderklinik, Düsseldorf Uni-Kinderklinik, Eberswalde Werner Forßmann Klinikum, Eckernförde Gem.-Prax, Eisleben Lutherstadt Helios-Klinik, Erfurt Kinderklinik, Erlangen Uni Innere Medizin, Erlangen Uni-Kinderklinik, Essen Diabetes-SPP, Essen Diabetes-Schwerpunktpraxis, Essen Elisabeth Kinderklinik, Essen Kinderarztpraxis, Esslingen Klinik für Kinder und Jugendliche, Esslingen Schwerpunktpraxis, Eutin Kinderklinik, Feldkirch Kinderklinik, Filderstadt Kinderklinik, Flensburg Diakonissen Kinderklinik, Forchheim Diabeteszentrum SPP, Frankenthal Kinderarztpraxis, Frankfurt Diabeteszentrum Rhein-Main-Erwachsenendiabetologie (Bürgerhospital), Frankfurt Diabeteszentrum Rhein-Main-pädiat. Diabetologie (Clementine-Hospital), Frankfurt Uni-Kinderklinik, Frankfurt Uni-Klinik Innere, Frankfurt-Höchst, Städtische Kinderklinik, Frankfurt-Sachsenhausen Innere, Frankfurt-Sachsenhausen Innere MVZ, Freiburg St. Josef Kinderklinik, Freiburg Uni Innere, Freiburg Uni-Kinderklinik, Freudenstadt Kinderklinik, Fulda Kinderklinik, Fürth Kinderklinik, Gaissach Fachklinik der Deutschen Rentenversicherung Bayern Süd, Garmisch-Partenkirchen Kinderklinik, Garmisch-Partenkirchen Klinikum Pädiatrie, Geislingen Klinik Helfenstein Innere, Gelnhausen Innere, Gelnhausen Kinderklinik, Gelsenkirchen Kinderklinik Marienhospital, Gera Kinderklinik, Gießen Ev. Krankenhaus Mittelhessen, Gießen Uni-Kinderklinik, Graz Uni Innere, Graz Uni-Kinderklinik, Greifswald Uni-Kinderklinik, Gummersbach Oberbergklinikum, Göppingen Innere Medizin, Göppingen Kinderklinik am Eichert, Görlitz Städtische Kinderklinik, Göttingen Uni Gastroenterologie, Göttingen Uni-Kinderklinik, Güstrow Innere, Hachenburg Kinderpraxis, Hagen Kinderklinik, Halle Uni-Kinderklinik, Hamburg Altonaer Kinderklinik, Hamburg Kinderklinik Wilhelmstift, Hamburg-Nord Kinder-MVZ, Hameln Kinderklinik, Hamm Kinderklinik, Hanau Kinderklinik, Hanau diabetol. Schwerpunktpraxis, Hannover DM-SPP, Hannover Kinderklinik MHH, Hannover Kinderklinik auf der Bult, Haren Kinderarztpraxis, Heide Kinderklinik, Heide Westküstenklinikum Innere Medizin, Heidelberg St. Josefskrankenhaus, Heidelberg Uni-Kinderklinik, Heidenheim Kinderklinik, Heilbronn Innere Klinik, Heilbronn Kinderklinik, Herdecke Kinderklinik, Herford Kinderarztpraxis, Herford Klinikum Kinder & Jugendliche, Heringsdorf Inselklinik, Herne Evan. Krankenhaus Innere, Hildburghausen Hennebergklinik, Hildesheim Bernward Krks Kinderheilkunde, Hildesheim GmbH - Innere, Hildesheim Kinderarztpraxis, Hildesheim Kinderklinik, Hof Kinderklinik, Hohenmölsen Diabeteszentrum, Homburg Uni-Kinderklinik Saarland, Idar Oberstein Schwerpunktpraxis, Ingolstadt Klinikum Innere, Innsbruck Uni-Kinderklinik, Innsbruck Universitätsklinik Innere, Itzehoe Kinderklinik, Jena Kinderarztpraxis, Jena Uni-Kinderklinik, Jena diabetol. Schwerpunktpraxis, Kaiserslautern Kinderarztpraxis, Kaiserslautern-Westpfalzklinikum Kinderklinik, Kamen Klinikum Westfalen Hellmig Krankenhaus, Kamen MKK - Medizinisches Kompetenzkollegium, Karlsburg Klinik für Diabetes & Stoffwechsel, Karlsruhe Schwerpunktpraxis, Karlsruhe Städtische Kinderklinik, Kassel Klinikum Kinder- und Jugendmedizin, Kaufbeuren MVZ für Kinder- und Jugendmedizin, Kempen Heilig Geist-KHS - Innere, Kempten Oberallgäu Kinderklinik, Kiel Städtische Kinderklinik, Kiel Universitäts-Kinderklinik, Kirchen DRK Krankenhaus Kinderklinik, Kirchheim-Nürtingen Innere, Klagenfurt Kinderklinik, Klagenfurt Klinikum am Wörthersee Abteilung IMuGast, Kleve Innere Medizin, Koblenz Kemperhof 1. Med. Klinik, Koblenz Kemperhof 1. Mediz. Klinik, Koblenz Kinderklinik Kemperhof, Konstanz Innere Klinik, Konstanz Kinderklinik, Krefeld Innere Klinik, Krefeld Kinderklinik, Kreischa-Zscheckwitz Klinik Bavaria, Köln Kinderklinik Amsterdamerstrasse, Köln Uni-Kinderklinik, Landau Innere, Landshut Kinderklink, Lappersdorf Kinderarztpraxis, Leer Klinikum - Klinik Kinder & Jugendmedizin, Leipzig Uni-Kinderklinik, Leoben LKH Kinderklinik, Leverkusen Kinderklinik, Lienz Diabetesschwerpunktpraxis für Kinder und Jugendliche, Limburg Innere Medizin, Lindlar DM-Zentrum, Linz KUK MedCampus IV Kinderklinik, Linz Krankenhaus der Barmherzigen Schwestern Kinderklinik, Lippstadt Evangelische Kinderklinik, Ludwigsburg Kinderklinik, Ludwigshafen Kinderklinik St.Anna-Stift, Lübeck Uni-Kinderklinik, Lübeck Uni-Klinik Innere Medizin, Lüdenscheid Märkische Kliniken - Kinder & Jugendmedizin, Lünen Klinik am Park, Magdeburg Ki-Klinik St. Marienstift, Magdeburg Städtisches Klinikum Innere, Magdeburg Uni-Kinderklinik, Mainz Uni-Kinderklinik, Manderscheid Rathauspraxis, Mannheim Uni-Kinderklinik, Marburg Uni-Kinderklinik, Marktredwitz Innere Medizin, Mechernich Kinderklinik, Meissen Kinderklinik Elblandklinikum, Memmingen Internistische Praxis, Memmingen Kinderklinik, Minden Kinderklinik, Moers Kinderklinik, Murnau am Staffelsee - diabetol. SPP, Mutterstadt Kinderarztpraxis, Mödling Kinderklinik, Mönchengladbach Kinderklinik Rheydt Elisabethkrankenhaus, Mühldorf am Inn Kinderarztpraxis, Mühlheim an der Ruhr Evang. Krankenhaus Med. Klin., München 3. Orden Kinderklinik, München Diabetes-Zentrum Süd, München Kinderarztpraxis diabet. SPP, München Praxiszentrum Saarstrasse, München von Haunersche Kinderklinik, München-Gauting Kinderarztzentrum, München-Harlaching Kinderklinik, München-Schwabing Kinderklinik, Münster Herz Jesu Innere, Münster Ludgerus-Kliniken GmbH, Münster St. Franziskus Innere Med., Münster St. Franziskus Kinderklinik, Münster Uni-Kinderklinik, Neuburg Kinderklinik, Neumünster Friedrich-Ebert-Kankenhaus Pädiatrie, Neunkirchen Gemeinschaftspraxis Kinderheilkunde, Neunkirchen Innere Medizin, Neunkirchen Marienhausklinik Kohlhof Kinderklinik, Neuruppin Kinderklinik, Neuss Lukas-Krankenhaus Kinderklinik, Neuwied Kinderklinik Elisabeth, Neuwied Marienhaus Klinikum St. Elisabeth Innere, Nidda Bad Salzhausen Klinik Rabenstein/Innere-1 Reha, Nürnberg Cnopfsche Kinderklinik, Nürnberg Uniklinik Med. Klinik 4, Nürnberg Uniklinik Zentrum f Neugeb./Kinder & Jugendl., Oberhausen Innere, Oberhausen Kinderklinik, Oberhausen Kinderpraxis, Oberhausen St.Clemens Hospitale Sterkrade, Oberwart - Burgenländische Krankenanstalten Pädiatrie, Offenbach/Main Innere Medizin, Offenburg Kinderklinik, Oldenburg Kinderklinik, Oldenburg Schwerpunktpraxis Pädiatrie, Olpe pädiatrische Gemeinschaftspraxis, Osnabrück Christliches Kinderhospital, Paderborn St. Vincenz Kinderklinik, Passau Kinderklinik, Pforzheim Kinderklinik, Pfullendorf Innere Medizin, Pirmasens Städtisches Krankenhaus Innere, Plauen Vogtlandklinikum, Prenzlau Krankenhaus Innere, Ravensburg Kinderklink St. Nikolaus, Regensburg Kinderklinik St. Hedwig, Remscheid Kinderklinik, Rendsburg Kinderklinik, Reutlingen Kinderarztpraxis, Reutlingen Kinderklinik, Reutlingen Klinikum Steinenberg Innere, Reutte Tirol BKH Kinderklinik, Rheine Mathiasspital Kinderklinik, Ried Innkreis Barmherzige Schwestern, Rosenheim Innere Medizin, Rosenheim Kinderklinik, Rosenheim Schwerpunktpraxis, Rostock Uni-Kinderklinik, Rotenburg/Wümme Agaplesion Diakonieklinikum Kinderabteilung, Rottweil Gemeinschaftspraxis für Innere Medizin, Rüsselsheim Kinderklinik, Rüsselsheim MVZ, Saaldorf-Surheim Diabetespraxis, Saarbrücken Kinderklinik Winterberg, Saarlouis Kinderklinik, Salzburg Universität Innere Medizin, Salzburg Universitäts-Kinderklinik, Scheibbs Landesklinikum, Scheidegg Prinzregent Luitpold, Schleswig Heliosklinik Kinderklinik, Schw. Gmünd Stauferklinik Kinderklinik, Schweinfurt Kinderklinik, Schwerin Innere Medizin, Schwerin Kinderklinik, Schwäbisch Hall Diakonie Kinderklinik, Siegen Kinderklinik, Singen Hegau Bodensee-Klinikum Kinderklinik, Singen Kinderarztpraxis, Spaichingen Innere, Speyer Diakonissen Stiftungskrankenhaus Pädiatrie, St. Augustin Kinderklinik, St. Johann Tirol Kinderklinik, St. Pölten Universitäts-Kinderklinik, St. Pölten Universitätsklinik Innere, Stade Kinderklinik, Steyr (Pyhrn-Eisenwurzen Klinikum), Abt. Kinder- und Jugendheilkunde, Stockerau Landeskrankenhaus, Stolberg Kinderklinik, Stuttgart Olgahospital Kinderklinik, Suhl Kinderklinik, Sylt Rehaklinik, Tettnang Innere Medizin, Traunstein Kinderklinik, Traunstein diabetol. Schwerpunktpraxis, Trier Kinderklinik der Borromäerinnen, Trostberg Innere, Tübingen Uni-Kinderklinik, Ulm Endokrinologikum Amedes, Ulm Uni Innere Medizin, Ulm Uni-Kinderklinik, Vechta Kinderklinik, Viersen Kinderkrankenhaus St. Nikolaus, Viersen internist. Praxis, Villach Kinderklinik, Villingen-Schwenningen Schwarzwald Baar Klinikum Kinderklinik, Villingen-Schwenningen Schwarzwald-Baar-Klinikum Innere, Volkertshausen Gemeinschaftspraxis, Vöcklabruck Kinderklinik, Waldshut Kinderpraxis, Wangen Oberschwabenklinik Innere Medizin, Waren-Müritz Kinderklinik, Weiden Kinderklinik, Weingarten Kinderarztpraxis, Weisswasser Kreiskrankenhaus, Wels Klinikum Pädiatrie, Wendelstein Kinder- + Jugendarztpraxis, Werningerode MVZ, Wesel Marienhospital Kinderklinik, Wien 3. Med. Hietzing Innere, Wien KH Nord-Klinik Floridsdorf, Wien Klinik Ottakring (Wilhelminenspital) 5. Med. Abteilung, Wien Preyersches Kinderspital, Wien Rudolfstiftung 1. Med. Abtl., Wien SMZ Ost Donauspital, Wien Uni Innere Med III, Wien Uni-Kinderklinik, Wiener Neustadt Landesklinikum Pädiatrie, Wiesbaden Helios Horst-Schmidt-Kinderkliniken, Wiesbaden Kinderklinik DKD, Wilhelmshaven Kinderarztpraxis, Wilhelmshaven Klinikum Kinderklinik, Winnenden Rems-Murr Kinderklinik, Witten Kinderarztpraxis, Wittenberg Innere Medizin, Wittenberg Kinderklinik, Wittlich DSP, Worms - Weierhof, Worms Kinderklinik, Worms Schwerpunktpraxis, Wuppertal Universitäts-Kinderklinik, Würzburg Kinderarztpraxis, Würzburg Uni-Kinderklinik, Zams Kinderklinik, Zweibrücken Kinderarztpraxis, Zwettl Landesklinikum Gmünd-Waidhofen, Zwettl Landesklinikum Gmünd-Waidhofen Kinderklinik.
